# The Ecological Design of Marine Urban Green Space Plant Landscaping Based on the Concept of Sustainability

**DOI:** 10.3390/plants13070923

**Published:** 2024-03-22

**Authors:** Jingwen Yuan, Chul Soo Kim

**Affiliations:** 1 Department of Marine Design Convergence Engineering, Pukyong National University, Busan 48513, Republic of Korea; 2 Department of Industrial Design, Pukyong National University, Busan 48513, Republic of Korea

**Keywords:** ocean city, ecological design, sustainable concept, green space, plant landscaping

## Abstract

With global climate change and accelerating urbanization, marine cities face unique environmental challenges. Ecological landscape creation is a form of design planning guided by the disciplines of landscape ecology and ecological aesthetics in the process of urban planning and construction. It seeks a design that can maintain the virtuous cycle of the ecosystem and at the same time maintain the spatial equilibrium of the dynamic development of urban landscapes, so as to make them have good ecological functions and corridor functions. The aim of this study is to explore the ecological design methods of plant landscaping in marine urban green spaces under the concept of sustainability. We first reviewed the concept of sustainable development and its application to urban green space design, especially the special requirements in the marine urban environment. This research focuses on how to select plant species that are adapted to the marine climate and how to promote biodiversity, enhance ecosystem services, and improve the quality of life of urban residents through eco-design approaches. Through the analysis of a number of domestic and international cases of green spaces in marine cities, we found that effective eco-design is not only about choosing the right plant species but also includes the rational management of water resources, soil protection, and ecosystem restoration, among other aspects. This study also points out that public participation and interdisciplinary cooperation play a crucial role in the ecological design process. Finally, this paper carries out a specific analysis of the landscape model landscape evaluation system for the ecological design of plant landscaping in marine urban green spaces and experimentally verifies that, compared with other styles, the experience of the European-style landscape is good overall. However, the view openness rating of the European style landscape is only about 0.42, and the best plant landscaping is the mixed mode of alkali poncho and salt poncho. This study aims to provide a practical reference and guidance for urban planners, landscape architects, and environmentalists.

## 1. Introduction

In the current context of global climate change and environmental degradation, sustainable development has become a core concept in urban planning and design. Sustainable development is one of the themes of today’s social development, and the construction and protection of the ecological environment of marine cities, as important places for human habitation, are particularly important. Green space plant landscaping is an important part of urban ecological design, which is of great significance for improving the urban environment and enhancing the quality of life of residents. This study is dedicated to exploring how to effectively realize the ecological design of green space planting in marine cities under the guidance of the concept of sustainable development, aiming to provide theoretical and practical support for the green transformation of cities.

In Section 2, this paper investigates and analyzes the literature prior research on the research topic and lists the findings of urban green space landscaping in some marine cities. This provides a necessary value reference for the subsequent research. Section 3 introduces the ecological approach to landscape design, introduces the concept of sustainability and the related concepts of planted landscaping in marine cities, and utilizes the SBE (scenic beauty estimation) method of evaluating the aesthetic quality of forest stands. This is a new assessment tool for measuring the performance of planted landscapes under the dual criteria of aesthetics and ecology. Section 4 presents a case study of ecological design practices to synthesize the results and discussion of this study. Section 5 then launches an exploration of future research directions.

This study adopts a multi-methodological research strategy with the aim of comprehensively exploring and evaluating the ecological design of planted landscapes in marine urban green spaces under the concept of sustainable development. First, Section 3.1, “Ecological Landscape Design Approaches under the Concept of Sustainable Development”, adopts a literature review approach. By reviewing and analyzing the existing literature, we constructed a theoretical framework for ecological landscape design, with special emphasis on the application of sustainable development concepts in planted landscapes in marine urban green spaces. In Section 3.2, the comparative research method is used to analyze the “ocean city”. By comparing the planting strategies of green spaces in different seaside climates, we explore the influence of various environmental factors on ecological design and propose adaptive design principles. In the analysis of “plant landscaping”, the elements of ecological design are analyzed from scientific and artistic perspectives. In Section 3.3, we summarize the need to integrate sustainability and phytoscape design in marine urban green spaces. In Section 3.4, we present the SBE stand aesthetic quality assessment methodology, a comprehensive assessment tool that combines quantitative and qualitative evaluation metrics to measure the ecological and aesthetic effectiveness of planted landscapes. In this study, this method was used to analyze and evaluate selected cases in depth. In Section 4, “Case Study”, five representative ecological designs of marine urban green spaces are compared and discussed. In Section 5, “Evaluation Analysis”, this study adopts a comprehensive analysis method, combining the findings and theoretical analyses of the previous chapters and drawing feasibility analyses from domestic and international eco-design cases. Finally, in Section 6, the significance and limitations of the findings are discussed, as well as the implications for future research.

Through this study, we hope to provide a comprehensive and in-depth theoretical framework for the ecological design of planted landscapes in marine cities’ green spaces and to demonstrate the possibility and effectiveness of sustainable landscape design in practical applications through empirical research. This is not only important for enhancing the quality of urban ecological environment but also contributes new perspectives and strategies for the sustainable development of marine cities.

## 2. Related Works

The ecological design of green space planting has long been studied by some experts, with Novriyanti, N. et al. taking Shanghai city as an example. They explored the mechanism of the influence of different green space characteristics on the physical activity behavior of the residents. They used the total amount of field physical activity as the dependent variable and urban greening attributes as the independent variables, and through logistic regression analyses, they found that there was a significant negative correlation between forest cover, scrub diversity, and plant diversity. This correlation applies to a wide range of fields, including ecological conservation, climate change response, and sustainable development. It also reveals the complex interdependencies and competitive mechanisms of plant growth, emphasizing the importance of the need to balance different vegetation levels in ecosystem management and conservation. Understanding and addressing this negative correlation is key to achieving biodiversity conservation and ecosystem health. In addition, there is a significant negative correlation between the road morphology index, green landscape visibility, and vegetation diversity [1]. In order to evaluate the effects of planting southern oak trees in unsuitable urban spaces, Kangwana, L. A. et al. proposed a sustainable and environmentally friendly approach for urban communities. They studied the effects of using these trees in urban spaces on their growth and general health. They used ArcGIS (Arc geographic information system) to obtain the GPS (geographic positioning system) coordinates of the trees and ArcMap to create a topographic map of the study area. The ArcMap 10.8 software has been used to determine the geographic location of the southern oak trees in the four study areas. This software was used to create a graphic profile of the four study areas and the East Baton Rouge area in which they are located. Eventually, the authors found that most of the southern oak trees had various problems because they were planted in the wrong urban space [2].

Zabihi, A. et al. used spatial syntactic techniques as a tool to explore the impact of different plant configurations on patient pathfinding. Their research focused on how implant design affects path selection by hospital users. In this process, the authors employed library research, Depthmap v.20 software from the University of London for computer simulation and analysis, and comparisons, among other methods. Their study demonstrated that different plant pairings have an effect on individual pathfinding behavior in healthcare environments. In addition to integrating the planted areas with the hospital architecture, a uniform and regular green design provides convenience and offers the possibility of pathfinding [3]. Belousova, O. et al. devised a landscape plan for the Eden Arboretum in Cornwall, England. In this analysis, the authors combined environmental, movement, historical, and cultural approaches, making it possible to identify various facilities within the arboretum that had universal cultural meanings. Through this plan, they were able to create a new, multi-purpose arboretum to transform the damaged land [4]. In order to solve the problem of large discrepancy in multidimensional urban landscape design, Liu, C. et al. proposed a multidimensional urban landscape design method based on nonlinear theory. Their simulation results showed that the mean value of the regression standard deviation was 0.567, the standard value was 0.753, and the F-test value was 0.655. Therefore, the visual characteristics of the multidimensional nonlinear landscape design using the proposed method were more expressive, and the landscape design effect was better [5]. 

There are two primary shortcomings of the existing green space plant landscaping. The first is focusing on aesthetics and ignoring the ecological adaptability and ecological function of plants, which leads to the likely death or loss of ecological function of plants and affects the ecological sustainability of the green space. The second is a lack of plasticity, in which the landscape cannot be adjusted and improved according to the actual situation of the green space, resulting in the ecological design of the green space lacking sufficient flexibility and efficacy.

## 3. Methods

### 3.1. Ecological Landscape Design Methods under the Concept of Sustainable Development

**Figure 1:**
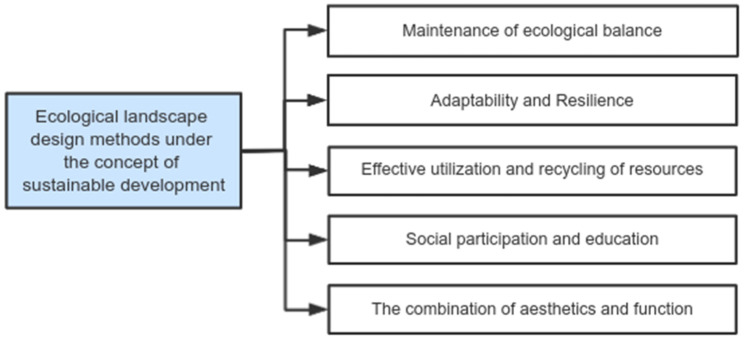
Concept of sustainable ecological development.

Sustainability is one of the core concepts of today’s social development. Especially in the field of urban and landscape design, the importance of this concept is becoming increasingly prominent. Ecological landscape design, under the concept of sustainable development, not only focuses on environmental protection and ecological balance but also emphasizes the harmonious coexistence of human activities and the natural environment. As shown in Figure 1, the following are a few key sustainability concepts:

Maintenance of ecological balance: Ecological landscape design emphasizes the maintenance and enhancement of the health and diversity of local ecosystems. This includes the use of native plants, the restoration of the natural environment, and the promotion of the flourishing of wild plants. The selection of plant species also requires an appropriate configuration based on local elements such as climate, topography, soil, and rainfall to establish a sustainable vegetation system. In the outdoor landscape design of different regions, it is necessary to plan for a reasonable arrangement of elements such as terrain, water, vegetation, and paving at the initial stages. Additionally, combining elements such as temperature variation, wind speed, rainfall, sunshine, and other factors in the region is crucial to reduce their negative impacts on the outdoor landscape and to improve people’s comfort in the outdoor landscape.

Adaptability and resilience: Considering climate change and environmental pressures, ecological landscape design needs to be adaptable and able to cope with future uncertainty and change. This includes the use of plants that can adapt to different environmental conditions and flexible spatial planning. Minimal interventions are taken in the planning and design stage to maximize the fit with the current characteristics of the site and to reflect the human characteristics of different areas [6,7]. On this basis, according to different uses, the landscape design should be divided into several functional areas to meet the needs of different groups.

Efficient use of resources and recycling: This concept encourages the use of sustainable materials and technologies, such as rainwater harvesting systems and solar lighting. It also advocates for the consideration of material recycling in the design stage to reduce waste and pollution. For hard materials such as concrete, stone, and wood, low-carbon and environmentally friendly materials are chosen to reduce energy consumption. Through the techniques of “infiltration, retention, storage, purification, and drainage”, the reuse of rainwater can be realized through the installation of permeable paving, sunken green space, and ecological grass-planting ditches, preventing waterlogging and forming a benign water-cycle system to achieve sustainable development [8].

Social participation and education: Sustainable landscape design involves both the creation of a physical space and a social practice. Through public participation, educational activities, and awareness raising, people can better understand and cherish the natural environment.

Combination of aesthetics and functionality: Sustainable landscapes should not only be eco-friendly but also aesthetically pleasing and functional. Designs should incorporate natural aesthetics to create spaces that are both comfortable and thought-provoking.

The concept of sustainability can be understood in three ways.

1. In order to continue to survive, people require necessities such as food, water, shelter, and so on. To achieve endless sustainable development, “We must commit ourselves to understanding the world, to understanding what is good for it, and to working with it and subjecting ourselves to it as it develops” [9].
2. A sustainable ecosystem is an earth-friendly, ever-improving state of the environment, that does not cause damage to the environment, as is the case with many human activities.
3. Solutions should be “people-centered” to achieve economical and environmentally friendly ecological development. Environmental problems do not exist in isolation, and most construction activities consume significant resources and land while at the same time bringing negative impacts to the surrounding environment and people’s lives. Therefore, the key to the design of planted landscapes is to be clear about what impact such changes will have on the local population [10].

### 3.2. Scope of Ocean Cities and Planted Landscapes

#### 3.2.1. Ocean Cities

Cities and towns are defined as “non-agricultural, animal husbandry” towns with a population mainly in the secondary and tertiary sectors. Cities and towns in China are set up according to the administrative divisions of the country. A marine city is a city consisting of a certain number of buildings on the sea that are capable of sustaining more than the standard number of local residents. The goal of marine construction and marine city construction is to maintain a comfortable living environment for people and to bring economic and social benefits to them, protecting and improving the ecological environment of the sea and the coast to promote the development of society [11].

As shown in Table 1, plant landscaping in marine cities generally involves the selection plants that are suitable for the climate of the seaside, such as beach plants, coastal plants, seaside trees, and marine herbs. These plants infuse the landscape of the marine city with the characteristics of the sea while also adapting to the special climate and environmental conditions of the seaside [12,13].

#### 3.2.2. Botanical Landscaping

Plant landscaping involves the use of trees, shrubs, vines, grasses, and other plants for landscape creation, which is inspired by nature’s plant communities and the imagery expressed. The plant community configuration in plant landscapes should be carried out in accordance with the laws of the development of plant communities in nature. If the selected plant species are not compatible with the environment and ecological conditions in which they are located, it is difficult for them to survive or grow, and they cannot meet the needs of the landscape creation. If a planting community structure is contrary to the natural community, it will not grow and develop the desired artistic effects [17]. Therefore, understanding the formation and development of natural plant communities, species compositions, structures, hierarchies and appearances is the basis for good plant landscape design [18]. A perfect plant landscape should achieve a high degree of consistency on both scientific and artistic levels, as shown in Table 2. This not only satisfies the consistency between plants and the environment in terms of ecological adaptability but also uses the principles of artistic composition to express the aesthetics of individual and groups of plants and the beauty of the context that is created by human beings in the process of viewing [19].

Plant landscaping should not only achieve the purpose of beautification but also improve the ecological environment of the city. The environment should be both scientific and artistic to create an ecological atmosphere that is integrated with nature. Ecological design is the most important method to create a plant landscape with spatial stability. By comprehensively analyzing the limiting factors that affect all kinds of ecological elements of the garden plant landscape, one can construct a layout model that maximizes its multiple functional effects [20].

### 3.3. The Need for Integration of Sustainability and Plant Landscape Design for Green Spaces in Marine Cities

The integration of sustainability in the design of planted landscapes for green spaces in marine cities is not only a trend but also a necessity. With the increasingly significant impacts of global climate change and rising sea levels, marine cities face unique challenges such as coastal erosion, salt water intrusion, and ecosystem destruction. Against this backdrop, integrating sustainability concepts into planted landscape design is essential to protect and enhance the ecosystems of marine cities and improve their resilience to climate change.

First, sustainable plant landscaping helps to enhance ecosystem services in marine cities. By selecting native plant species that are salt- and wind-resistant, the natural resilience of ecosystems can be enhanced, providing habitats for wildlife and maintaining biodiversity. In addition, these plants improve the microclimate of the city by providing necessary shade and cooling effects, thereby improving the quality of life for city residents.

Second, sustainable design supports the efficient management of water resources. Marine cities often face a shortage of freshwater resources. Measures such as rainwater harvesting and utilization and the creation of natural wetlands can not only reduce dependence on freshwater resources but also effectively control flooding and reduce urban runoff. These measures help to maintain the balance of the water cycle and improve the self-sufficiency of cities.

Furthermore, sustainable plant landscaping helps enhance community cohesion and environmental awareness. Through public participation, residents can be directly involved in the planning and maintenance of planted landscapes, thereby increasing their awareness of and participation in environmental protection. This sense of participation and identity is crucial to the establishment of a sustainable community culture.

In addition, sustainable plant landscaping is an important contribution to urban aesthetics. By creating diverse and attractive green spaces, it not only enhances the visual aesthetics of cities but also provides places for recreation and entertainment, improving mental health and quality of life.

Finally, sustainable plant landscaping is also an important means of addressing future challenges. As the uncertainty of climate change increases, flexible and adaptable planting can better cope with possible future environmental changes such as extreme weather events and rising sea levels.

In summary, integrating sustainability concepts into the design of green space planting in marine cities not only enhances the ecological quality of the city and the quality of life of its residents but also improves the city’s resilience in the face of environmental challenges, making it a key strategy for the sustainable development of marine cities.

### 3.4. Evaluation Methodology

#### 3.4.1. SBE Method

The SBE method is a method of evaluating the aesthetic quality of a forest stand. Color photographs are used to represent the landscape of a forest stand, and a slide show is used as the evaluation medium to ascertain the public’s preference for the forest stand in 3 steps.

Shooting and preparing color photos: First, representative forest stand landscapes are selected and high-quality color photos are taken. These photos should show the characteristics of the forest stand in a comprehensive way, including the types, sizes, and forms of the trees, as well as elements such as understory vegetation and forest open spaces. The photographs should be selected with consideration of the landscapes during different seasons and times of day (e.g., morning, evening) to show the aesthetic characteristics of the forest stand under different conditions.

Assembling a slide show and designing an evaluation questionnaire: The selected color photographs should be made into a slide show format that is ready to be used in a subsequent evaluation meeting or survey. At the same time, an evaluation questionnaire containing criteria should be designed for evaluating the aesthetic quality of each slide, including a survey of preferences which may include questions on the sense of naturalness, harmony, diversity, etc., of the landscape.

Organize evaluation activities and collect data: One or more evaluation sessions should be organized, and participants should be invited from different backgrounds (e.g., forestry experts, local residents, tourists, etc.) to view the slides and fill out the questionnaire. In this way, direct feedback from the general public on the aesthetic quality of the forest stand is collected. The collected data are then analyzed to understand which forest stand features are the most popular and which may need improvement.

Through these three steps, the SBE method enables an effective evaluation the aesthetic quality of forest stands, obtaining direct feedback from the public on their landscape preferences for forest stands and providing a scientific basis for forest stand management and planning. This approach places a special emphasis on public participation and multifaceted perspectives, helping to improve the overall aesthetic and ecological value of forest stand landscapes.

#### 3.4.2. Multi-Task Joint Design Process Modeling Study Using the SBE Method and Image Descriptions

A multi-task joint design process modeling study employing the SBE method (scene-based estimation) along with image descriptions involves the simultaneous generation of scene understanding and image descriptions using deep learning techniques. The basic principle of this approach is to combine the tasks of recognizing and describing image contents and process them through a unified framework to improve the overall performance and efficiency of the system. The components are multi-task learning, scene understanding, image description generation, joint optimization, and cooperation between attention mechanism and feature fusion. In the SBE method, scene understanding and image description generation are considered related tasks, and both tasks are learned simultaneously by sharing certain layers in a deep neural network. The scene elements in the image are recognized and understood, and in the multi-task joint design, the generation of image descriptions will utilize the deep semantic information provided by the scene understanding task. The two tasks promote each other and jointly improve the performance of the model, ensuring that the generated descriptions not only accurately reflect the image content but are also rich and detailed.

**Figure 2:**
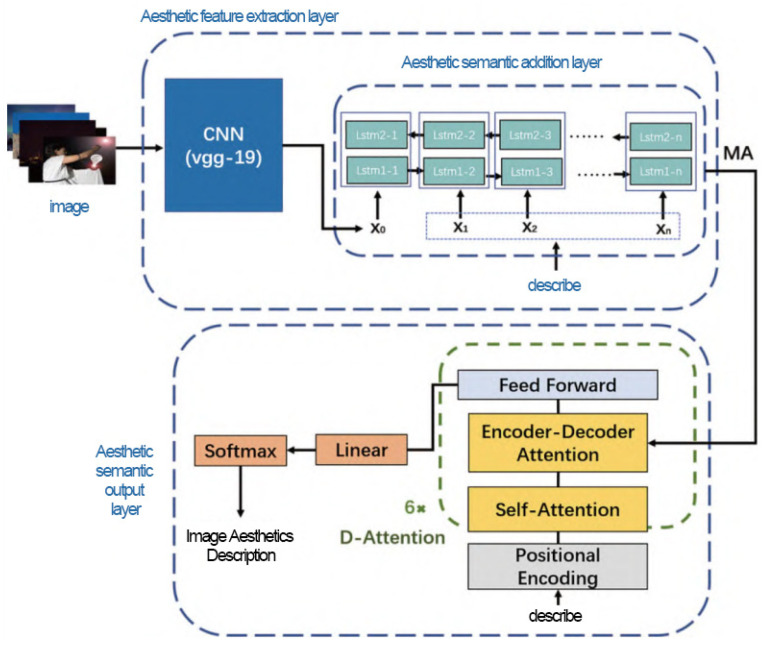
Diagram of DIAReviewer network structure.

Shasha Lv [18] introduced the residual network idea on the basis of the VGG-19 model and introduced the spatial attention mechanism and the channel attention mechanism. They also proposed the deep image aesthetic reviewer (DIAReviewer) model, which realizes the combination of the two tasks of the evaluation of the aesthetic quality and the description of the image. The network structure of the DIAReviewer is shown in Figure 2. The image goes through an aesthetic feature extraction layer to extract information, attach it to a description layer of aesthetic semantics, and output a description result regarding the aesthetics of the image.

## 4. A Case Study of Ecological Design for Planted Landscapes in Marine Urban Green Spaces

### 4.1. Case Selection Criteria and Analysis Methods

For the study of the ecological design of plant landscaping in marine urban green spaces, wanting to realize its application with sustainable concepts, firstly, green space parks with typical characteristics at home and abroad were chosen to analyze and research. These were chosen to understand the ecosystems of plant types, water resources environments, soil protection, ecosystem restoration, and other dimensions, and to provide a clear picture of the ecological design. This chapter will study, summarize, and draw conclusions based on the design process and technical realization of the application of plant landscaping ecological design in the green spaces of marine cities.

The case study of this research involves the design of the planted landscapes in urban green spaces in marine cities. The case locations are selected from a total of five domestic and international seaside cities, shown in Table 3, including Republic of Korea, Singapore, the Netherlands, Denmark, and Spain. The above sources of ecological information regarding urban green spaces in marine cities include a literature survey and seaside landscape survey.

### 4.2. Ecological Case Studies of Planted Landscapes in Marine Urban Green Spaces

Five representative cases of marine urban green spaces were ecologically analyzed based on plants, water resources, soil, and ecosystems to be interpreted separately. Table 4 shows the ecological analysis of the urban green space for Han River Park in Seoul. The vegetation type in the Hanjiang River Basin is a subtropical evergreen broadleaf forest. Through the influence of climatic factors, subtropical evergreen broadleaf forests are evergreen year-round and generally dark green. The forest phase is neat, and the forest canopy is microwave-like undulation. The height of the community is generally about 15–20 m, rarely more than 30 m, and the total degree of depression is 0.7–0.9. The following summary is drawn from the investigation of the current ecological data of the park.

Table 5 shows the ecological analysis of the urban green space for the Marina Bay Gardens. The highlighted area within the Gardens by the Bay is the Supertree Grove. The Supertree Grove is a forest of 18 tree-like structures ranging in height from 20 to 50 m. Each tree is covered with a variety of climbing plants, epiphytes, and ferns, forming a vertical garden. The tops of the giant trees are equipped with photovoltaic cells that absorb solar energy during the day and illuminate them at night, while the crowns of the trees act as exhaust vents that are connected to the plant greenhouses, mimicking the photosynthesis and respiration of trees. At night, the Giant Tree Forest holds regular light show performances that are colorful and spectacular. The following summary is derived from a survey of the park’s current ecological data.

As shown in Table 6, an ecological analysis of urban green spaces was conducted for Amstel Park. The park has a true tri-climate greenhouse that is divided into three zones with their own temperature, humidity, and air circulation. Visitors can walk through a South African bush, dry desert, and tropical jungle. Located in the tropics, it is home to ancient palms and thuja trees. Along with 23 ancient or rare trees, there is a collection of carnivorous and medicinal plants. The following summary is derived from a survey of ecological information on the current state of the park.

Table 7 shows an ecological analysis of urban green spaces for Habler Park. Denmark has a temperate oceanic climate with abundant rainfall and hot and humid conditions, resulting in a continuous, green, lush, multi-layered, dense jungle of herbs, vines, and epiphytes. Many plants try to grow upwards in order to obtain more sunlight. The following summary is derived from a survey of the park’s current ecological data.

Table 8 provides an ecological analysis of urban green spaces in Barcelona. Barcelona has a Mediterranean climate with four distinct seasons that are hot and dry in the summer and mild and rainy in the winter. The design of the park is dedicated to the vegetative characteristics of the Mediterranean region, taking into account the topography of the site and the local climatic influences. The Barcelona Botanical Garden combines Mediterranean landscapes, fractal geometric compositions, and materials with carefully chosen colors, all of which make it a microcosm of the Mediterranean landscape that is reminiscencent of the Mediterranean agrarian landscape. The following summary is derived from a survey of the current ecological data of the park.

### 4.3. Analytical Summary

As shown in Table 9, based on the results of the characterization of the five main cases, a comparative analysis was carried out in which plant species, water management, soil protection, and ecosystem restoration were all summarized separately.

Seoul’s Han River Park, Singapore’s Gardens by the Bay, Amstel Park in Amsterdam, Copenhagen’s Habule Park, and Barcelona’s Seaside Green demonstrate diverse strategies and practices for the ecological design of green spaces in different marine cities. Seoul’s Han River Park focuses on the diversity of native plants and the use of natural rainwater, emphasizing eco-engineering techniques to facilitate the restoration of riverbank ecosystems. In contrast, Singapore’s Gardens by the Bay capitalizes on its tropical setting, combining native and exotic plants to create ecological diversity and enhance ecosystem services through efficient water recycling systems and eco-engineering techniques. Amstel Park in Amsterdam and Habule Park in Copenhagen also emphasize native plant species and natural water management, but while Amstel Park focuses more on wetland ecosystem restoration and ecological connectivity, Habule Park enhances soil health and ecological balance through sponge city principles and organic farming methods.

Barcelona’s waterfront green spaces, on the other hand, show how water management and soil conservation can be optimized through plant species adapted to Mediterranean environments and water-saving techniques. It also emphasizes organic soil amendments and natural erosion control measures. Overall, these cases reflect a preference for native and adapted plant species in the ecological design of urban green spaces. They also provide as a common focus on stormwater management and soil conservation techniques. They each employ strategies that are appropriate for their particular climatic, cultural, and environmental needs, demonstrating the important role of ecological design in enhancing urban biodiversity, improving environmental quality, and increasing the well-being of residents.

In marine urban green spaces, ecological design is important for promoting sustainable urban development and ecological restoration, especially in special environments such as marine cities, where biodiversity is essential for maintaining ecological balance and improving ecosystem resilience. By studying successful cases, it is possible to learn how to effectively integrate green spaces and urban environments to realize eco-services such as rainwater management, air quality improvement, temperature regulation, etc. This knowledge provides the necessary background to guide future sustainable urban planning and design. In addressing climate change, the role of green space eco-design in mitigating and adapting to climate change was emphasized, such as reducing the urban heat island effect by increasing the urban green cover and using waterfront green spaces as a natural barrier to mitigate the impacts of rising sea levels and extreme weather events. In terms of improving the well-being of residents, it demonstrated how the creation of an environment that is conducive to the physical and mental health of residents, including the reduction of noise pollution, the provision of space for recreation and sports, and the enhancement of community connectivity, can directly improve the quality of life of residents. In terms of promoting technological and methodological innovations, the analysis emphasizes the role of green space eco-design in mitigating and adapting to climate change. It also stimulates innovations in new technologies and methods, such as smart irrigation systems, eco-restoration techniques, and the integrated application of green infrastructure. In terms of strengthening social and cultural values, it reveals how green space eco-design can reinforce local characteristics and cultural identity, deepening residents’ sense of identity and belonging to their living environment through the protection and display of native plant species and historical landscapes.

## 5. Evaluation and Analysis of the Ecological Design of Plant Landscaping in Urban Green Spaces

Six coastal towns within domestic and international marine cities such as Busan, Qingdao, and Singapore were selected as case studies and were evaluated in conjunction with aquatic plant landscaping patterns. Referring to the previous research results, the landscape beauty level is divided into the following four levels: extremely unattractive, unattractive, average, and beautiful. The corresponding scores are 0, 1, 2, and 3, respectively, and the evaluation results are shown in Table 1.

### 5.1. SBE Evaluation System

The SBE evaluation system was modified to establish a suitable landscape quality evaluation system, and the evaluation criteria and scoring basis are shown in Table 10.

The different modes of plant landscaping include the natural landscape mode, modern landscape mode, Japanese landscape mode, and European landscape mode. Each of these patterns has its own characteristics and is suitable for different scenarios and design needs. The characteristic scores of four planted landscaping style patterns in six coastal towns within domestic and international marine cities, including Busan, Qingdao, and Singapore, were derived from 100 questionnaires and ranked and compared from 0.0 to 1.0. Eight indicators including environmental safety; environmental accessibility; environmental cleanliness; environmental order; plant species, colors, and seasonal variations; vegetation ratio; vegetation growth status; and vegetation morphology were determined.

**Figure 3:**
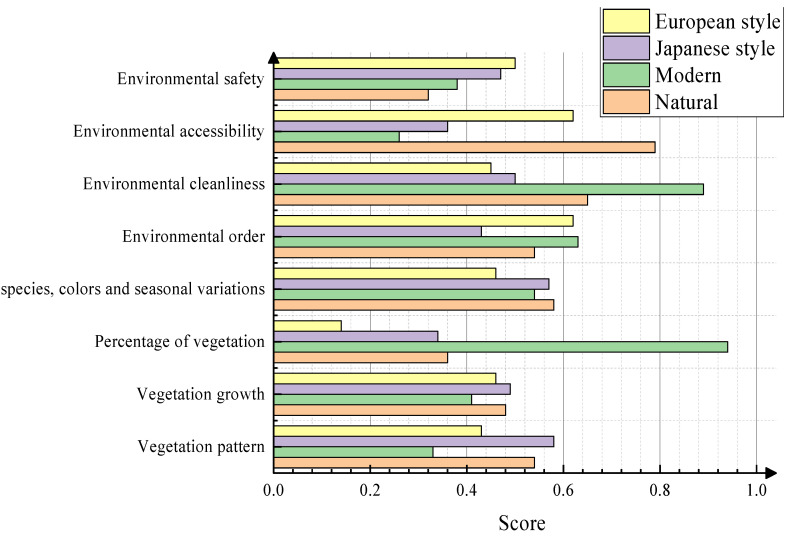
Landscape scores for environment and vegetation.

As can be seen from Figure 3, the score of the modern landscape based on the eight indicators of environmental safety; environmental accessibility; environmental cleanliness; environmental order; plant types, colors, and seasonal changes; vegetation proportions; vegetation growth conditions; and vegetation form was poor. The indicator with the lower score was environmental accessibility, with a score of approximately 0.242 points. The highest evaluation index of the modern landscape was the proportion of vegetation, with a score of approximately 0.92. The European-style, Japanese-style, and natural-style landscapes had relatively low scores based on the eight landscape characteristics evaluation indicators. The scores for the eight evaluation indicators of the Japanese-style landscapes were lower than 0.6, failing to reach a passing score. Therefore, the sample data regarding the landscapes of ocean cities need to be improved.

**Figure 4:**
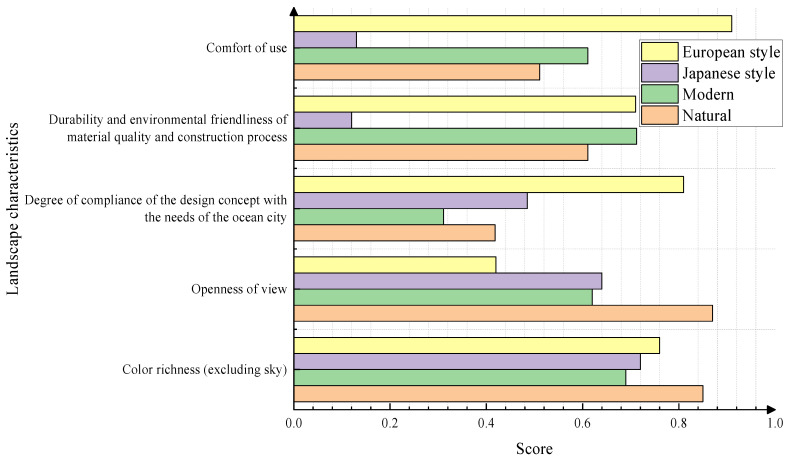
Ratings of the experiential aspects of the landscape.

The experiential characteristics were divided into five parts under the four plant landscaping style patterns: comfort of use, durability and environmental friendliness of the material quality and construction process, fit of design concepts with the needs of the ocean city, openness of view, and richness of color. The results of the questionnaire survey were analyzed. As can be seen from Figure 4, compared with the other styles, the European-style landscape experience was generally good. However, the field of view score of the European-style landscape was only about 0.42. The comfort of Japanese-style landscaping as well as the durability and environmental friendliness of the material quality and construction technology were poor, with scores of only about 0.12. However, this landscape received a score of 0.72 in terms of its color richness. The overall rating of the natural landscape was acceptable, but some of the landscape design concepts were not in line with the needs of the ocean city. The maximum score was 3 points. Based on the data reported in Figure 3 and Figure 4, the existing ocean city landscape needs to be improved. 

### 5.2. Beauty Value of Different Configuration Modes

The results of the evaluation of the beauty of the three seasons of spring, summer, and autumn in nine configuration modes and their rankings according to the average value of the beauty of the three seasons are shown in Figure 3.

Calculation method to determine the scenery’s beauty value: The research results show that the weighted summation method has certain rationality for the evaluation of the scenery’s beauty. Therefore, the weighted summation method is used for the calculation of the scenery’s beauty in this article. The calculation formula is as follows:

(1) $$S_{i}=10\times \sum_{j=1}^{10}\left(\frac{n_{ij}}{N}\times j\right)$$

In the formula, $S_{i}$ is the beauty value of photo *i*, *N* is the total number of people who evaluated it, and $n_{ij}$ is the number of people who gave a *j*-score to photo *i*. 

**Figure 5:**
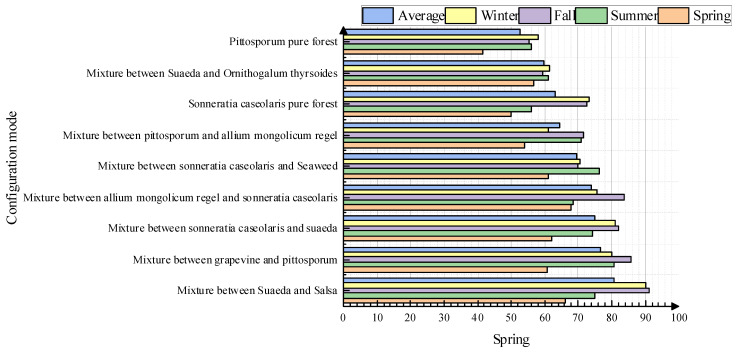
Evaluation values of landscape beauty in spring, summer, autumn, and winter for nine configuration modes.

In order to improve the design of plant landscaping in ocean cities, this article explores different plant configuration modes that are consistent with the environmental survival of ocean cities with the aim of optimizing the current ecological design of ocean city landscapes. Figure 5 shows the aesthetic scores of the seasonality among the 9 configuration patterns. The aesthetics of the different landscaping designs shown in Figure 5 are sorted according to their average score. The landscaping of pure pittosporum forest cannot meet the landscaping principles of seasonal changes and diversified tones, so this configuration model has the lowest score. The best plant landscaping is a mixture of Suaeda and Salsa. 

## 6. Discussion

### 6.1. Research Innovations and Significance

First, through an in-depth analysis of the existing literature, this study constructs a theoretical framework on ecological landscape design. This framework particularly emphasizes the application of the concept of sustainable development in plant landscaping in marine urban green spaces and provides new theoretical guidance for the design of marine urban green spaces. Second, this study adopts a comparative research methodology to explore the influence of environmental factors on ecological design by analyzing the plant landscaping strategies of green spaces in different seaside climates. It also proposes adaptive design principles that provide specific operational guidelines for the ecological design of marine urban green spaces. In addition, this study introduces the SBE stand aesthetic quality assessment method, a new assessment tool for comprehensively measuring the performance of planted landscapes under the dual criteria of aesthetics and ecology. The application of this method not only enriches the assessment toolbox for green space design in marine cities but also provides a new evaluation perspective in the field of landscape design.

In addition, this study’s exploration of an ecological approach to landscape design under the concept of sustainable development is of great significance in promoting the innovation and development of planted landscapes in marine urban green spaces. It not only promotes the enhancement of ecological protection and biodiversity but also raises people’s awareness of the importance of a sustainable living environment. It provides a comprehensive assessment framework for the ecological design of marine urban green spaces, combining quantitative and qualitative evaluation indicators and enabling designers to better understand and assess the ecological and aesthetic effects of planted landscapes. Through the comparative discussion in the case study section, this study not only reveals the strengths and limitations of different design strategies but also provides an empirical basis for future marine urban green space design. Finally, the findings and theoretical analyses are summarized through a comprehensive analytical approach. This study provides new ideas and strategies for the ecological design and sustainable development of marine urban green spaces, and it is instructive for future research directions in related fields.

### 6.2. Research Limitations and Prospects

The feasibility of this study is mainly attributed to its adoption of a multi-methodological research strategy. This not only permits an in-depth discussion of the ecological design of planted landscapes in marine urban green spaces from different perspectives but also enhances the comprehensiveness and depth of the study through the integrated use of a literature review, comparative research methods, SBE forest stand aesthetic quality assessment methods, and case studies. In particular, by introducing the SBE stand aesthetic quality evaluation method, this study was able to comprehensively evaluate the ecological and aesthetic effects of planted landscaping on both quantitative and qualitative bases, which provided new assessment tools and perspectives for the design of green spaces in marine cities. In addition, through case studies, this study not only demonstrates the practical application of different design strategies but also compares and analyzes the strengths and limitations of each case, thus providing an empirical basis and reference for future ecological design practice.

However, this study also has certain limitations. First, although a multi-methodological strategy was adopted, the number of cases studied is limited and may not be able to fully represent the diversity of green space design in marine cities around the world. Secondly, although the SBE forest stand aesthetic quality evaluation method provides a new tool for the assessment of planted landscapes, the application of this method may be limited by the subjective judgment of the evaluator, which affects the objectivity of the evaluation results. In addition, this study mainly focuses on theoretical construction and case study analyses, with less exploration of the specific problems and challenges that may be encountered during practical operation and implementation.

To address the above limitations, future research directions can be developed based on the following aspects. First, the scope of the case study can be expanded to include more cases of marine urban green space designs in more countries and regions to improve the universality and representativeness of the study. Secondly, future research can further improve and optimize the SBE forest score aesthetic quality evaluation method, increase the objective evaluation indexes, and reduce the influence of subjective judgment. In addition, future research can also pay more attention to the implementation process and effect assessment of ecological design, exploring how to overcome the challenges of design and implementation in practice and how to effectively monitor and assess the long-term effects of ecological design after implementation. Finally, in view of global challenges such as climate change and accelerated urbanization, future research should also explore how the design of green spaces in marine cities can be integrated with the overall sustainable development strategy of the city to achieve wider ecological, social, and economic benefits.

## 7. Conclusions

With the changes in social structures and conditions in China, the continuous improvements in people’s living standards, the continuous acceleration of urbanization, the continuous expansion of urban areas into the suburbs and even to the countryside, the diversion of rivers, the re-planning of lakes and green areas, etc., the present designs of marine cities sometimes fail to satisfy the problems of marine environmental protection and coastline planning. The design of urban landscapes is related to the work, life, leisure, and entertainment of urban residents. Therefore, it is important to consider people’s livelihoods when designing these projects. Applying urban concepts to urban landscape designs ensures that the city meets the needs of its citizens in their daily lives. This is achieved by alleviating the water cycle problems brought about by urbanization, providing an indispensable transformation and implementation plan in the sustainable development of urban planning. This paper analyzes the different landscapes in various cities, summarizes the specific schemes of landscape transformation based on the design concepts of sponge cities, and coordinates the balance between water cycle protection and landscape aesthetics based on the specific design scheme of the urban landscape. This provides a complete perspective on the diversification of landscape design based on the goal of not destroying the ecological balance. In general, at the same time, economic development cannot ignore the structure of the biological chain. To comply with the core idea of sponge city theory, the design of the urban landscapes should be in line with the working life of the public while not destroying the water system cycle.

## Figures and Tables

**Table 1:** Plants suitable for seaside climates.

Botanical Classification	Growing Environment	Plant Name	Photograph
Beach plant	Adapted to sandy soils and sea breezes	Sea vegetables, sand grapes, sand onions, etc.	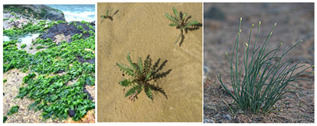
Shoreline plant	Seawater-soaked and saline environments [14]	Some salt-tolerant plants, such as alkali pongo, alkali pongo grass, and salt pongo	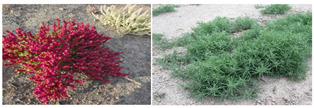
Beach trees	Suitable for growing environments on the seashore, resistant to sea wind erosion and salt erosion [15]	Sea mulberry, sea tongue, Sea mulberry elm, etc.	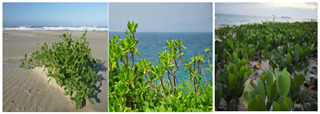
Marine herbs	In humid environments, providing important ecological functions [16]	Seaweeds, sea onions, etc.	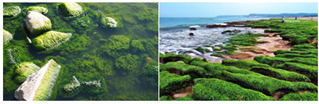

**Table 2:** Analysis of plant layout elements.

Level	Realm	Characterization	Key Constituent	Element
The scientific dimension	Vegetative	Ecological consistency	Temperature	Plants adapt to specific temperature ranges by regulating physiological processes such as photosynthesis and respiration, allowing different species of plants to thrive in specific climate zones.
			(fig.) overstatement	Plants are adapted to the water conditions of their environment, responding to dry or wet conditions through mechanisms such as root uptake, leaf transpiration, and intracellular water regulation.
	Environment		Sunlight	Plants adjust their photosynthetic efficiency and growth habits according to the intensity and quality of available light, thus surviving and reproducing in different light environments.
			Atmosphere	Plants regulate gas exchange through the opening and closing of stomata, adapting to different air qualities and carbon dioxide concentrations to optimize photosynthesis and water use.
			Ground	Plants adapt their root structure and nutrient uptake strategies to different soil environments based on soil texture, pH, nutrient content, and microbial communities.
The artistic dimension	Vegetative	Aesthetic consistency	Permutation	The arrangement of plants, such as symmetrical or asymmetrical and natural or elaborate, can create visual fluidity and rhythm, bringing harmony and dynamic beauty to the environment.
			Coloration	The color diversity of plants, from bright flowers to deep green leaves, provides rich layers of color and emotional expression to the environment, adding vividness and energy.
	Environment		Framework	The structural forms of plants, such as slender blades of grass, meandering vines, and hard trunks, provide a wealth of textures and shapes that inspire artistry and design.
			Appearances	The overall appearance of the plants, including their shape, size, and attitude, can influence their visual impact on the environment, creating a sense of natural beauty and elegance that harmonizes or contrasts with their surroundings.

**Table 3:** Ecological park cases.

No	Park Name	Nations
A	Seoul Han River Park	Republic of Korea
B	Gardens by the Bay	Singaporean
C	Amsterdam Park	The Netherlands
D	Habrer Park	Denmark
E	Barcelona Riviera Green	Spanish

**Table 4:** Seoul Han River Park.

Outline	The planting of diverse native plants and the creation of ecological wetlands strengthens the city’s ecological network and enhances biodiversity. It provides its residents with a recreational space that is close to nature and demonstrates a model of ecological restoration and sustainable development in the city.
Photograph	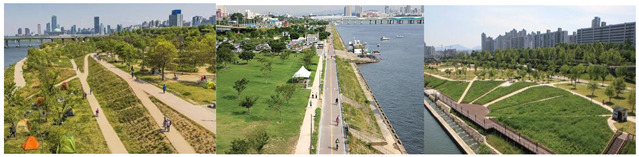
Plant species	Diverse native plants, including perennial herbs and native tree species, and vegetation unique to riparian ecosystems.
Water management	Natural rainwater harvesting and filtration systems are used, as well as natural purification from riparian vegetation.
Soil protection	Use of eco-engineering methods such as vegetative cover and natural erosion control measures to minimize soil erosion.
Ecosystem restoration	Rebuild the natural landscape of the riverbanks, enhance biodiversity, and strengthen the function of the ecological corridor.

**Table 5:** Gardens by the Bay.

Outline	Through the integration of an innovative eco-design and sustainable technologies, such as mega artificial trees to promote air purification as well as water recycling systems and eco-wetlands, the city’s ecological infrastructure has been strengthened, biodiversity has been enhanced, and the garden has become a unique green eco-artistic landscape in the city.
Photograph	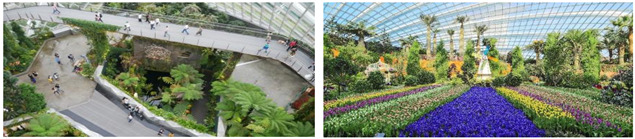
Plant species	Tropical plants and sustainable ecological landscapes that combine native and exotic plants to create diverse ecological environments.
Water management	Efficient water recycling systems, including rainwater harvesting and recycling and water features.
Soil protection	Utilize ecological engineering and plant cover techniques to improve soil quality and reduce erosion.
Ecosystem restoration	Create multi-layered ecological spaces that promote biodiversity and provide a wildlife habitat.

**Table 6:** Amstel Park.

Outline	Amstel Park’s urban green space showcases ecological diversity through its unique tri-climate greenhouse, providing a cross-climatic travel experience from the South African bush and desert to the tropical jungle. This design not only increases the richness of plant species but also provides visitors with opportunities to educate and explore different ecosystems. By maintaining ancient trees and rare plants, the park emphasizes the importance of ecological conservation and sustainability.
Photograph	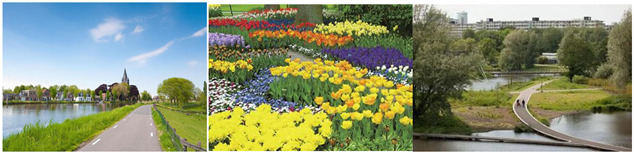
Plant species	Focus on planting native native plants, as well as wetland plants that are adapted to the waterside environment.
Water management	The park has a network of water systems, as well as a system for the natural collection and filtration of rainwater.
Soil protection	Use natural vegetation cover and ecological engineering techniques to maintain soil health and stability.
Ecosystem restoration	Restore the original wetland ecosystem and enhance biodiversity and ecological connectivity.

**Table 7:** Habrer Park.

Outline	By extensively planting native plants and utilizing eco-friendly design principles, this park restores the natural ecology at the edge of the city’s watershed and enhances the biodiversity of the area. At the same time, it provides citizens with a green space that integrates natural beauty and recreational activities, demonstrating a successful practice of sustainable ecological development in the city.
Photograph	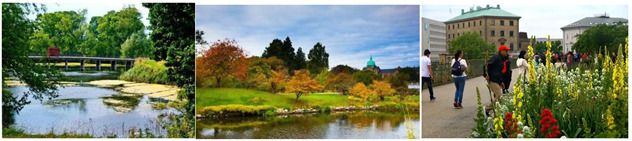
Plant species	Emphasis is placed on Nordic native plants, including diverse herbs and hardy trees.
Water management	Effective management of rainwater and surface water using sponge city principles, i.e., the use of urban structures to absorb, store, infiltrate, and purify rainwater in order to cope with urban flooding and improve the water environment.
Soil protection	Organic farming and natural soil management practices are used to maintain the ecological balance of the soil.
Ecosystem restoration	Focus on restoring seaside ecosystems and upgrading urban green infrastructure.

**Table 8:** Barcelona Waterfront Green Space.

Outline	By restoring and expanding native plant populations and creating multi-functional green spaces and ecological corridors, the city not only enhances its biodiversity and green coverage but also improves the urban microclimate and provides its residents with a pleasant environment for recreation and interaction, reflecting the close integration of urban ecosystem management and sustainable development.
Photograph	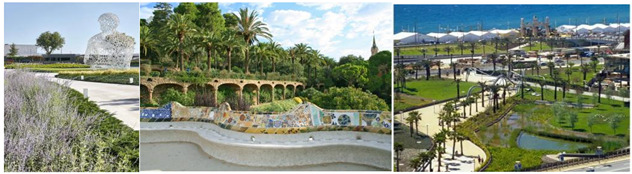
Plant species	Combination of Mediterranean plants and salt-tolerant plants for seaside environments.
Water management	Adoption of water-saving irrigation systems and rainwater recycling techniques.
Soil protection	Emphasize organic soil amendments and natural erosion control measures.
Ecosystem restoration	Restoration of riparian vegetation, enhancement of biodiversity, and utilization of native and adapted plant species have strengthened ecosystem stability and resilience.

**Table 9:** Comparative analysis of green space ecological design cases in marine cities.

No	Case	Plant Species	Water Management	Soil Protection	Ecosystem Restoration
A	Seoul Han River Park	Herbaceous and native species	Collection and filtration systems	Resist corrosion	Promoting biodiversity
B	Gardens by the Bay	Tropical plants	Water circulation system	Resist corrosion	Promoting biodiversity
C	Amstel Park	Wetland plants	Collection and filtration	Organic food	Promoting biodiversity
D	Habrer Park	Herbaceous plants and hardy trees	Irrigation and recycling	Organic food	Upgrading urban green infrastructure
E	Barcelona Riviera Green	Mediterranean and salt-tolerant plants	Irrigation and recycling	Resist corrosion	Enhancing ecosystem stability and resilience

**Table 10:** Measured landscape characteristics.

Landscape Characteristic	Score			
	0	1	2	3
Environmental order	Messy	General	Neat	
Environmental cleanliness	Dirty	Average	Clean	
Vegetation pattern	All artificial vegetation	Mostly artificial vegetation	Mix of plants for artificial and seaside climates	Most or all of the plants for a seaside climate
Vegetation growth	Poor	Average	Better	Very good
Proportion of vegetation	Unsuitable	More suitable	Suitable	Very well suited
Plant species, colors, and seasonal changes	Single species	2–5 species of plants	Seasonal plants of 2–5 species	Seasonal, color-diverse plants of 2–5 species
Environmental accessibility	Inaccessible	Harder to reach	Average	Easily accessible
Environmental safety	Dangerous	Average	Safe	Very safe
Color richness (excluding sky)	1 type	2 species	3 species	4 or more species
Visual openness	Enclosed	Average	Open	
Conformity of the design concept to the needs of an ocean city	Does not meet	Average	Conforming	Perfect for needs
Durability and environmental friendliness of material quality and construction process	Not durable or environmentally friendly	Durable but not environmentally friendly	Environmentally friendly but not durable	Durable and environmentally friendly
Comfort of use	Not comfortable	General	Comfortable	Very comfortable

## Data Availability

The raw data supporting the conclusions of this article will be made available by the authors, without undue reservation.
